# Prediction of GABARAP interaction with the GABA type A receptor

**DOI:** 10.1002/prot.25589

**Published:** 2018-11-04

**Authors:** B.W.J. Irwin, Siniša Vukovič, M.C. Payne, Mohammad ElGamacy, P.‐L. Chau

**Affiliations:** ^1^ Department of Physics Theory of Condensed Matter Group, Cavendish Laboratory, University of Cambridge Cambridge United Kingdom; ^2^ Abteilung Proteinevolution Max‐Planck‐Institut für Entwicklungsbiologie Tübingen Germany; ^3^ Bioinformatique Structurale, CNRS URA 3528 Paris France

**Keywords:** free energy change, GABA_A_ receptor, GABARAP

## Abstract

We have performed docking simulations on GABARAP interacting with the GABA type A receptor using SwarmDock. We have also used a novel method to study hydration sites on the surface of these two proteins; this method identifies regions around proteins where desolvation is relatively easy, and these are possible locations where proteins can bind each other. There is a high degree of consistency between the predictions of these two methods. Moreover, we have also identified binding sites on GABARAP for other proteins, and listed possible binding sites for as yet unknown proteins on both GABARAP and the GABA type A receptor intracellular domain.

## 1 INTRODUCTION

The GABA_A_‐receptor associated protein, GABARAP, was first described by Wang et al.^1^ It is a protein of 117 amino acids and has a relative molecular mass of 13 900. These authors also determined that it interacted with amino acids 394‐411 of the intracellular domain of the *γ*2‐subunit of the GABA_A_ receptor. If this sequence was shortened to 399‐411 or 389‐402, then the interaction was no longer observed. These authors also reported that GABARAP 36‐117 and GABARAP 1‐68 both interacted with the *γ*2‐subunit in the GST pull‐down assay, indicating that the interaction domain spanned GABARAP amino acids 36‐68. In a subsequent paper, Nymann‐Andersen et al.^2^ concluded that the octadecapeptide RTGAWRHGRIHIRIAKMD from the GABA_A_ receptor *γ*2‐subunit was necessary and sufficient for interacting with the GABARAP, but the interaction, as determined by the glutathione‐*S*‐transferase pull‐down assay, was not as high as that given by the tricosapeptide CFEDCRTGAWRHGRIHIRIAKMD. This molecule gave the highest level of activity in the assay.

Knight et al.^3^ examined the NMR shift of the GABARAP cross‐peaks when the octadecapeptide RTGAWRHGRIHIRIAKMD was present. They noticed that the NMR signals from GABARAP amino acids Val 31, Arg 40, Asp 45, Lys 46, Leu 50, Val 51, Leu 55, Thr 56, Phe 60, Ile 64, Arg 65 and Glu 101 were significantly changed, with Lys 46, Val 51, Phe 60 and Ile 64 displaying changes of the order of 1 linewidth. These authors also estimated the dissociation constant of the octadecapeptide RTGAWRHGRIHIRIAKMD from GABARAP to be higher than 0.2 mM, so the measured binding was weak.

Coyle et al.^4^ measured intrinsic tryptophan fluorescence to study the binding between GABARAP and the *γ*2‐subunit of the GABA_A_ receptor. They used native GABARAP, GABARAP with the first 10 amino acids truncated (*Δ*N10) and GABARAP with the first 27 amino acids truncated (*Δ*N27). They found that the dissociation constant between the octadecapeptide RTGAWRHGRIHIRIAKMD and native GABARAP was 1.29 ± 0.09 *μ*M, between the octadecapeptide and *Δ*N10 was 1.17 ± 0.06 μM, and between the octadecapeptide and *Δ*N27 was 6.10 ± 0.29 μM. The dissociation constant between native GABARAP and the tridecapeptide RTGAWRHGRIHIR was 3.33 ± 0.34 μM, and between native GABARAP and the undecapeptide GAWRHGRIHIR was 5.52 ± 0.52 μM. These dissocation constants are much smaller than that determined from NMR by Knight et al.,^3^ and it is still unclear where the source of the large discrepancy lies.^5^

The function of GABARAP is most probably 2‐fold: anchoring the GABA_A_ receptor to the cytoskeleton, and modulating the function of the receptor. Amino acids near the N‐terminal of GABARAP could bind to tubulin,^4^ while the amino acids nearer the C‐terminal bind to the GABA_A_ receptor.^2^ Chen et al.^6^ showed that GABARAP caused GABA_A_ receptor clustering, and clustered receptors exhibited lower affinity for GABA (EC_50_ increased from 5.74 ± 1.4 μM to 20.27 ± 3.8 μM), and they desensitized less quickly (the desensitization time constant *τ* increased from 1 second to 2 seconds). Everitt et al.^7^ performed electrophysiology experiments and showed that GABARAP promotes the clustering of GABA_A_ receptors, and increases the conductance of the GABA_A_ receptor from below 40 pS to above 50 pS.

Despite all these advances on the interaction between the GABA_A_ receptor and GABARAP, we still do not know the structural details of this interaction. Weiergräber et al.^5^ cocrystallized GABARAP with the K1‐peptide (sequence DATYTWEHLAWP) and determine the structure to 1.3‐Å resolution. They used these data and previous published data to infer the interaction between GABARAP and the GABA_A_ receptor.

In this work, we used experimental structures of the GABARAP and a modeled structure of the intracellular domain of the GABA_A_ receptor, and performed docking simulations. We also carried out simulations of the docked structures. Independently, we also used inhomogeneous fluid solvation theory (IFST)^8^, ^9^ to calculate the free energy of displacing all reasonable clusters of water containing 7‐18 molecules from the surface of the intra‐cellular domain of the GABA_A_ receptor, and from the surface of experimental structures of GABARAP. This information was applied to validate the docking interaction between the GABA_A_ receptor and GABARAP, in the context of surface hydration following the methods of Vukovič et al.^10^

## 2 METHODS

### 2.1 Molecular coordinates

In this research, we used the coordinates of a GABA_A_ receptor model from the work of Mokrab et al.^11^ This model used as template the nicotinic acetylcholine receptor (nAChR) structure from the work of Unwin,^12^ where five intracellular helices were resolved (Protein Data Bank code: 2BG9). Thus, this is the only model of the GABA_A_ receptor that includes part of the intracellular domain. The subunit composition of this receptor is (*α*1)_2_(*β*2)_2_*γ*2.

There exist five stand‐alone structures of GABARAP, and their Protein Data Bank codes are, respectively, 1GNU, 1KJT, 1KOT, 1KLV, and 1KM7. 1GNU and 1KJT come from X‐ray crystallography experiments, and we chose 1GNU because of its higher resolution of 1.75 Å. 1KOT, 1KLV, and 1KM7 all come from NMR experiments; 1KM7 contains only one conformer, while residues 1‐17 in 1KLV could not be located and so we chose 1KOT with 15 conformers. We thus used two structures of GABARAP. One is an NMR solution structure, PDB code 1KOT,^13^ and the other is an X‐ray crystallography structure, PDB code 1GNU.^3^

### 2.2 Docking

There are 15 slightly different conformations in the NMR structure 1KOT. They will hereafter be called 1KOT model 1 to 1KOT model 15. The X‐ray structure 1GNU contains only one coordinate set, but Ser 16, Ser 53, and Arg 65 have been resolved with two alternative conformations, each with occupancy 1/2. We thus generated eight structures from the 1GNU coordinate set, each with slightly different conformations. They will hereafter be called 1GNU‐aaa to 1GNU‐bbb, depending on whether the A‐form or the B‐form from the Protein Data Bank was chosen.

Twenty‐three coordinate sets, 15 from NMR experiments, and eight from X‐ray crystallography experiments, were used as the ligand for SwarmDock.^14^, ^15^ This docking method allows for flexibility of the molecules using normal modes,^16^ and the use of the program is available on a public server (as of the time of writing this article, the SwarmDock server resides on https://bmm.crick.ac.uk/svc-bmmswarmdock/index.html). For the receptor, we used the modeled coordinates of the tricosapeptide C^420^FEDCRTGAWRHGRIHIRIAKMD^442^ from the *γ*2‐subunit of the GABA_A_ receptor; this is the section from Cys 420 to Asp 442. Experiments by Nymann‐Andersen et al.^2^ showed that this tricosapeptide gave full binding to GABARAP. We had tried docking GABARAP to the complete GABA_A_ receptor, but this was rejected by SwarmDock as the GABA_A_ receptor contained too many atoms (14 900 nonhydrogen atoms). Therefore we used only part of the *γ*2‐subunit in the docking. In this work, we did not specify the interface amino acids and only used ‘blind’ docking. A maximum of five normal modes were allowed for each molecule.

SwarmDock produced 468 docks for each GABARAP conformation. The output consisted of 10 764 coordinates of different conformations of GABARAP and the tricosapeptide from the GABA_A_ receptor. The coordinates of the latter were slightly different from the original tricosapeptide coordinates, as SwarmDock flexible docking has changed the structure of both the receptor and the ligand. We used a least‐squares fit to superimpose the SwarmDock structure of the receptor onto the original tricosapeptide coordinates; the translation vector and rotation matrix used were noted. The same vector and matrix were subsequently used to move GABARAP to a model of the complete GABA_A_ receptor whose *γ*2 tricosapeptide position were coincident with that of the tricosapeptide used in the docking. We then tested for steric clashes between GABARAP and the GABA_A_ receptor. If two atoms, one from each protein, were found to be within 1 Å of each other, that dock was rejected.

The results filtered for steric clashes were then selected using the following criteria:

1. At the interface, the GABARAP amino acids Lys 46, Val 51, and Phe 60 were all present.
2. At the interface, at least one of the GABA_A_ receptor amino acids Arg 425, Thr 426, Gly 427, Ala 428, or Trp 429 was present.
3. At the interface, at least one of the GABA_A_ receptor amino acids Arg 433, Ile 434, His 435, Ile 436, Arg 437, Ile 438, Ala 439, Lys 440, Met 441, or Asp 442 was present.

Criterion 1 was applied to locate docking positions consistent with NMR experiments.^3^ In this paper, Ile 64 was also identified as an important interface amino acid, but its position means that we were unable to obtain any docking poses with Ile 64 at the interface. Criteria 2 and 3 were applied to extract docks consistent with the yeast two‐hybrid assay.^1^ 161 docks were selected after these procedures.

We undertook further filters to select the optimal docks from these 161 docks: we examined the distribution of these 161 docks according to the following seven criteria:

- The SwarmDock energy score should be in the more favorable half of the energy score distribution.
- The number of ligand amino acids with at least one atomic contact to the receptor amino acids Arg 425 to Trp 429 and Arg 433 to Asp 442 should be in the higher half of the corresponding distribution.
- The number of ligand amino acids with at least one atomic contact to the “cytoplasmic” receptor amino acids Arg 425 to Trp 429 should be in the higher half of the corresponding distribution.
- The number of ligand amino acids with at least one atomic contact to the “membrane” receptor amino acids Arg 433 to Asp 442 should be in the higher half of the corresponding distribution.
- The number of receptor amino acids with at least one atomic contact to any ligand amino acid should be in the higher half of the corresponding distribution.
- The number of atomic contacts from the ligand to any of the receptor amino acids Arg 425 to Trp 429 and Arg 433 to Asp 442 should be in the higher half of the corresponding distribution.
- The number of atomic contacts from the receptor to any ligand amino acids should be in the higher half of the corresponding distribution.

In the above criteria, a contact was defined as an atom which was less than the sum of the van der Waals radii of the two atoms +20%.^14^, ^15^ A dock was selected from these 161 configurations if all of these additional seven criteria were met.

These seven additional criteria were chosen to enforce that the best ligand structure should have a competitive energy score such that the structure is stable (criterion 4), maintain an overall high contact to the receptor (criterion 5) to multiple sites which are distributed between the upper (criterion 6) and lower (criterion 7) portions of the receptor sequence. The best structures must also reciprocate contact across many sites on the ligand (criterion 8) and the strength of all contacts should be a close and strong as possible on the receptor (criterion 9) and ligand (criterion 10).

### 2.3 Simulation of GABARAP and intracellular helices

**Figure 1:**
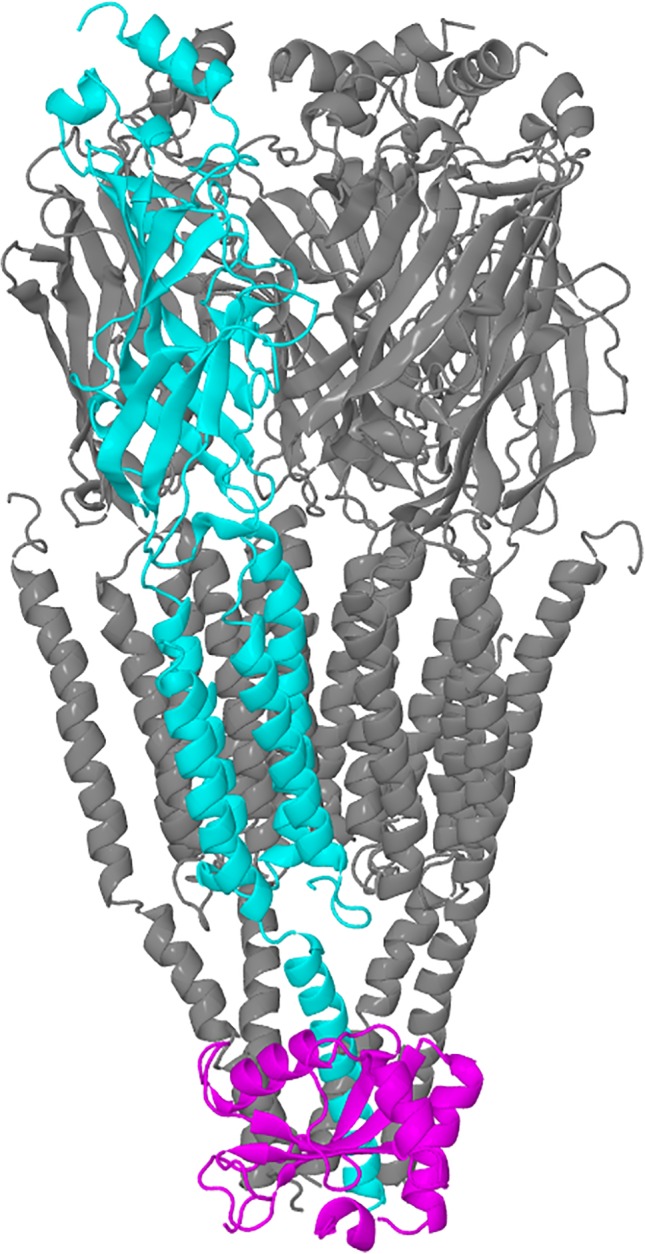
Diagram showing a model of the GABA_A_ receptor and a proposed docking pose of the GABARAP, 1KOT model 1 dock 17d. The GABA_A_ receptor is modeled using 2BG9 as the template, and so only part of the intracellular domain is modeled. The *γ*2‐subunit is shown in cyan, and the rest of the receptor shown in gray; GABARAP is shown in magenta [Color figure can be viewed at wileyonlinelibrary.com]

We took two representative docked structures of GABARAP and three intracellular helices of the GABA_A_ receptor, and performed simulations on these complexes. Figure 1 shows the docking of GABARAP to the GABA_A_ receptor.

**Figure 2:**
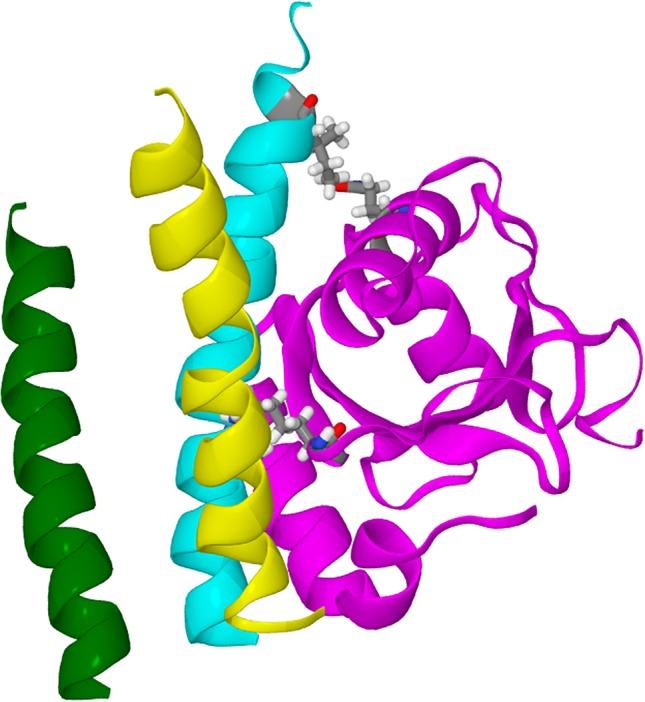
Diagram showing a model of the GABA_A_ receptor and a proposed docking pose of the GABARAP, 1KOT model 15 dock 54a. Only part of the GABA_A_ receptor is shown in this diagram. GABARAP is shown in magenta, and the interaction intracellular helix of the *γ*2‐subunit is shown in cyan. The *α*1‐subunit intracellular helix is shown in yellow, and the *β*2‐subunit helix is shown in green. At the membrane end of the *γ*2‐subunit helix, asp 423 interacts with GABARAP Lys 46. At the cytoplasmic end of the helix, Ile 438 interactis with GABARAP Gln 59 [Color figure can be viewed at wileyonlinelibrary.com]

The two docked structures chosen were 1KOT model 15 dock 54a and 1GNU bbb‐conformer dock 41d. Each structure consisted of GABARAP in the docking position beside the intracellular helix of the *γ*2‐subunit of the GABA_A_ receptor, from Asp 413 to Asp 442, together with the intracellular helices of the two adjacent subunits. They were included to provide a more realistic environment for GABARAP. These two helices comprised the *α*1‐subunit of the GABA_A_ receptor from Lys 391 to Ser 417, and the *β*2‐subunit from His 421 to Thr 444. The GABARAP/trihelix complex is shown in Figure 2.

The GABARAP/trihelix complexes were placed in a periodic box with at least 10 Å between the protein and its image. The system with the 1KOT model consisted of 17 406 water molecules, 49 K^+^ ions and 61 Cl^−^ ions to achieve a [KCl] of 0.15 mM. The system comprised a total of 55 655 atoms. The system with the 1GNU model consisted of 17 985 water molecules, 51 K^+^ ions and 63 Cl^−^ ions to achieve a [KCl] of 0.15 mM. The system comprised a total of 57 396 atoms.

Each system was minimized for 10 000 steps with all the protein atoms frozen. Molecular dynamics was initialised for 10 000 time‐steps of 0.1 fs each, with all main‐chain nitrogen atoms frozen. Langevin dynamics was applied; the thermostat was set with a time constant of 1 ps^–1^, and the barostat set with a piston decay time of 10 ps and a piston period of 20 ps. The van der Waals cut‐off was 12 Å, and Ewald summation was used for long‐range electrostatics. The time‐step was lengthened to 2 fs over 30 000 time‐steps, while all main‐chain nitrogen atoms of the three helices were tethered with a force constant of 2 kJ/mol/Å^2^. A 40‐ns equilibration was carried out on the initialised system, followed by a data collection period of 100 ns. Configurations were output every 2 ps.

We calculated the r.m.s. deviation of the simulated structures from the original starting structure. We also evaluated the distance between the *γ*2‐subunit helix Asp 423 and GABARAP Lys 46, and *γ*2‐subunit Ile 438 and GABARAP Gln 59. As can be seen in Figure 2, Asp 423 is at the membrane end of the intracellular helix, and Ile 438 is at the cytoplasmic end of the helix. We monitor these distances to see if GABARAP stays bound to the GABA_A_ receptor throughout the simulations.

### 2.4 Free energy change calculations

The molecules were prepared using the CHARMM‐GUI freely available on the web (as of the time of writing this article, the address of the CHARMM‐GUI is http://charmm-gui.org).^17^ The molecular dynamics package NAMD 2^18^ was used in this work.

In the simulation of the intracellular helices of the GABA_A_ receptor we first selected atoms from the following amino acids: *α*1‐subunit Lys 391‐Leu 422, *β*2‐subunit His 421‐Ile 449 and *γ*2‐subunit Asp 413‐Ser 443. The helices were placed in a periodic box with at least 10 Å between the protein and its image. The system consisted of 19 708 water molecules, 56 K^+^ ions and 73 Cl^−^ ions to achieve a [KCl] of 0.15 mM. The system comprised a total of 61 857 atoms.

The system was minimized for 10 000 steps with all the protein atoms frozen. Molecular dynamics was initialised for 10 000 time‐steps of 0.1 fs each, with all main‐chain nitrogen atoms frozen. Langevin dynamics was applied; the thermostat was set with a time constant of 1 ps^‐ 1^, and the barostat set with a piston decay time of 10 ps and a piston period of 20 ps. The van der Waals cut‐off was 12 Å, and Ewald summation was used for long‐range electrostatics. The time‐step was lengthened to 2 fs over 30 000 time‐steps, while all main‐chain nitrogen atoms were frozen. A 2‐ns equilibration was carried out on the initialised system. A data collection simulation was then carried out for 5 ns, again with all main‐chain nitrogen atoms fixed. Configurations were output every 0.5 ps. We obtained a total of 10 000 configurations of the intracellular helices of the GABA_A_ receptor.

For the simulation of GABARAP, we chose model 3 of 1KOT and the 1GNU structure (AAA) as the starting structures. The 1KOT structure of 117 amino acids was placed in a periodic box with at least 10 Å between the protein and its image; 9161 water molecules, 24 K^+^ ions and 26 Cl^−^ ions were placed in this box. The system consisted of a total of 29 508 atoms. The 1GNU structure of 117 amino acids was placed in a periodic box with at least 10 Å between the protein and its image; 9115 water molecules, 25 K^+^ ions and 27 Cl^−^ ions were placed in this box. The system consisted of a total of 29 372 atoms.

These systems were minimized for 10 000 steps with all main‐chain nitrogen atoms frozen. Langevin dynamics was applied; the thermostat was set with a time constant of 1 ps^‐ 1^, and the barostat set with a piston decay time of 1 ps and a piston period of 2 ps. The van der Waals cut‐off was 12 Å, and Ewald summation was used for long‐range electrostatics. The time‐step was lengthened to 2 fs over 40 000 time‐steps. The system was then equilibrated for 2 ns. Data collection was carried out for 5 ns, again with all main‐chain nitrogen atoms frozen, with configurations output every 0.5 ps. We obtained a total of 10 000 configurations for each model of the hydrated GABARAP.

The MD trajectory for the GABA_A_ receptor was processed as described by Vukovič et al.^10^ First, hydration sites as defined by Haider and Huggins^19^ were created on all surface regions of the GABA_A_ receptor. The hydration sites were time averaged water molecules assigned positions, densities and occupancies.^20^, ^21^ Hydration sites with a radius of 1.2 Å were picked starting from the densest patch of water in order of decreasing density and no sites were picked within 2.4 Å of an already existing site. Next, an IFST calculation for the free energy was carried out for each of the hydration sites according to IFST described in Vukovič et al.^10^ IFST had previously been used on water molecules around proteins where the proteins are involved in binding small ligands^22^, ^23^, ^24^ and in protein–protein interactions.^25^ All 10 000 snapshots of the protein sampled at 0.5 ps intervals were used to calculate the free energy difference associated with hydrating each site with a single water molecule. These free energy differences were mostly negative because solvation was favorable.

At this stage some hydration sites were removed to improve the efficiency of the combinations algorithm. Hydration sites inside the ion channel of the GABA_A_ receptor were removed; the ion channel was aligned to the *z*‐axis, the positions of all protein atoms were converted to cylindrical coordinates with a height *z*, and a radius and angle in the *xy*‐plane. The cylindrical mid‐plane of the protein atoms as a function of height and averaged over angle was found by fitting a quadratic polynomial to the protein atom data. Hydration sites on the inside of this mid‐plane were removed. Hydration sites with coordinate *z* >  − 48 Å were also removed as this region was close to the lipid bilayer in the full GABA_A_ receptor model.

Then a combinatoric search scheme was employed to search for up to the best 1000 clusters containing from 7 to 18 hydration sites within 12.5 kJ/mol of the best cluster. The search was run three times with these parameters, the first time searching for “near” clusters with hydration sites at most 3.1 Å away from nonhydrogen atoms and 3.6 Å away from hydrophobic nonhydrogen atoms, the second time searching for “regular” clusters with hydration sites at most 3.6 Å away from nonhydrogen atoms and 4.1 Å away from hydrophobic nonhydrogen atoms, as originally performed by Vukovič et al.^10^ The third search was for “far” clusters with hydration sites at most 4.1 Å away for nonhydrogen atoms and 4.5 Å away for hydrophobic nonhydrogen atoms. These three ranges were selected to observe how the hydration patches changed on variation of the hydration site cutoff distance from the protein that is, the degree to which bulk‐like distal waters are included in hydration patches.

The method used by Vukovič et al.^10^ predicts ligandability of drug molecules to a protein, and advances in combinatoric search allow clusters of this size to be found. These authors conclude that, for a small peptide, clusters of 30 hydration sites may need to be considered. Finding clusters with volumes commensurate with the ligand in this case is computationally infeasible, especially as GABARAP is much larger than a small peptide. As the free energy change of displacing hydration sites relative to bulk water atoms tends to zero at distances as small as 7 Å‐8 Å from the surface,^10^ one could instead search for a *clustering of clusters* with the most favorable displacement free energy scores to estimate candidate regions for larger objects to bind, namely proteins. This method was employed for the GABA_A_ receptor. The set of hydration sites within the best 1000 clusters for each size of 7 to 18 hydration sites were filtered, and turned into hydration patch data for all three classes of clusters, “near,” “regular” and “far.” For GABARAP, multiple “regular” passes were made of the hydration patch combinatoric search, and after each iteration, the hydration sites associated with patches identified previously were removed. There were 5 passes for the 1KOT file and 4 passes on the 1GNU file, after which no more sites could be found. The first‐pass sites take the least energy to displace and hence are the most displaceable and the fifth‐pass ones are the least displaceable.

## 3 RESULTS

### 3.1 Docking

**Figure 3:**
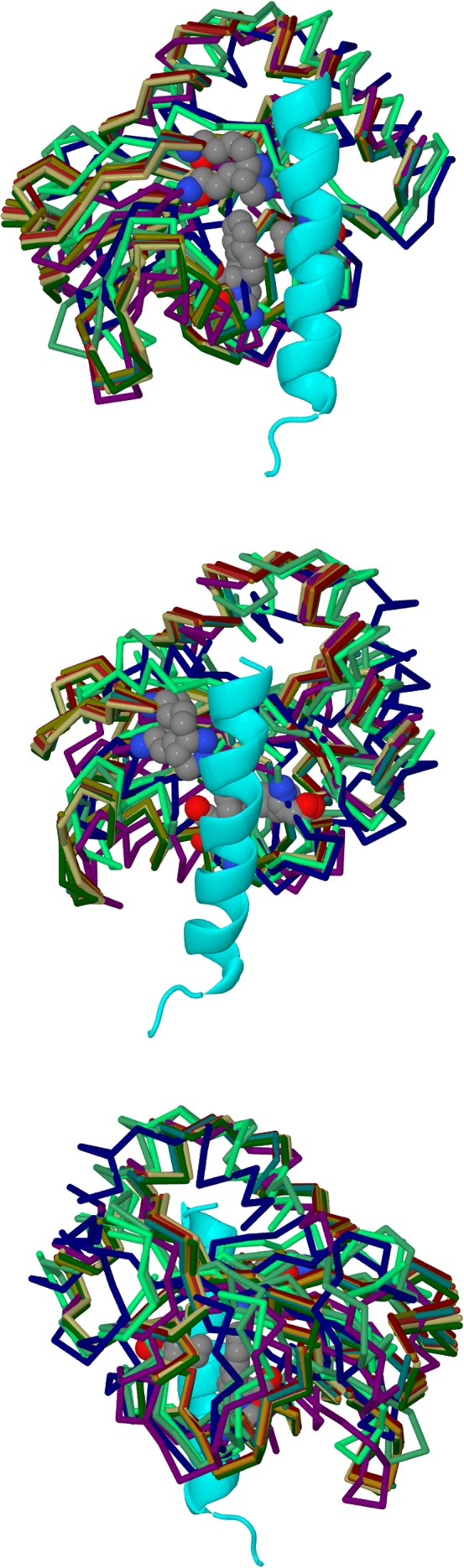
Diagrams showing the 11 proposed docks; they were selected from the SwarmDock results, according to criteria from experiments. The three panels show alternative views of the docking. A section of the *γ*2‐subunit is shown in cyan, and the 11 docked poses of GABARAP shown in different colors. GABARAP amino acids Lys 46, Val 51 and Phe 60 are highlighted in space‐filling models colored according to atom identity. The extracellular space is toward the upper part of the diagram. In the top and middle panels, the angle of view is from the ion channel toward the outside of the receptor. In the bottom panel, the angle of view is from outside the receptor toward the ion channel [Color figure can be viewed at wileyonlinelibrary.com]

SwarmDock produced 10 764 docks, and 161 docks were selected according to the first three criteria described in the previous section. Using seven additional criteria, we identified 11 docks, two of them coming from 1GNU and nine from 1KOT. The configuration of these docks are shown in Figures 1 and 3, and the coordinates are deposited in supplementary material. These configurations show a high degree of similarity between all 11 docks. The root‐mean‐square deviation of C*α*‐atoms between all 11 docks was calculated and is shown in Table 1. The largest deviation in the structure comparisons was 2.57 Å, between 1GNU‐bbb dock 41d and 1KOT model 1 dock 17d.

**Table 1:** The 11 chosen docks were: (1) 1GNU‐aaa dock 28b, (2) 1GNU‐bbb dock 41d, (3) 1KOT model 1 dock 17d, (4) 1KOT model 11 dock 29d, (5) 1KOT model 15 dock 39b, (6) 1KOT model 15 dock 40c, (7) 1KOT model 15 dock 40d, (8) 1KOT model 15 dock 41a, (9) 1KOT model 15 dock 42c, (10) 1KOT model 15 dock 54a, (11) 1KOT model 15 dock 54d. The following table shows the root‐mean‐square deviation between these 11 structures in Å. Column 1 and row 11 have been omitted due to redundancy

Dock	2	3	4	5	6	7	8	9	10	11
Num										
1	0.67	2.56	2.36	2.36	2.42	2.35	2.35	2.35	2.35	2.35
2		2.57	2.40	2.40	2.47	2.39	2.40	2.39	2.38	2.56
3			1.69	1.62	1.65	1.60	1.58	1.68	1.60	1.60
4				1.55	1.54	1.53	1.51	1.56	1.53	1.54
5					0.34	0.17	0.19	0.18	0.11	0.08
6						0.41	0.41	0.41	0.38	0.38
7							0.09	0.17	0.14	0.14
8								0.22	0.15	0.16
9									0.20	0.19
10										0.06

In Table 2, we list the contacts between amino acids pairs, one from each protein. Some of these contacts have few contact atoms and are only observed in one docked pair. Other contacts have many contact atoms, and are found in all 11 docked pairs. In this table, we only list contact pairs where there are more than 10 contact atoms, and where they are observed in at least nine out of the 11 docked poses.

**Table 2:** Table showing the contact pairs between the receptor and the ligand, and the frequency of finding that contact pair

GABA_A_‐R amino acid	GABARAP amino acid	Freq. Of occurrence
Asp 423	Lys 46	11/11
Cys 424	Leu 50	11/11
Ala 428	Leu 50	11/11
Ala 428	Arg 28	11/11
Arg 430	Arg 67	11/11
Ile 434	Leu 63	11/11
Ile 438	Gln 59	11/11
His 431	Leu 63	10/11
His 435	Gln 59	10/11
Cys 420	Lys 48	9/11
His 431	Tyr 49	9/11

**Figure 4:**
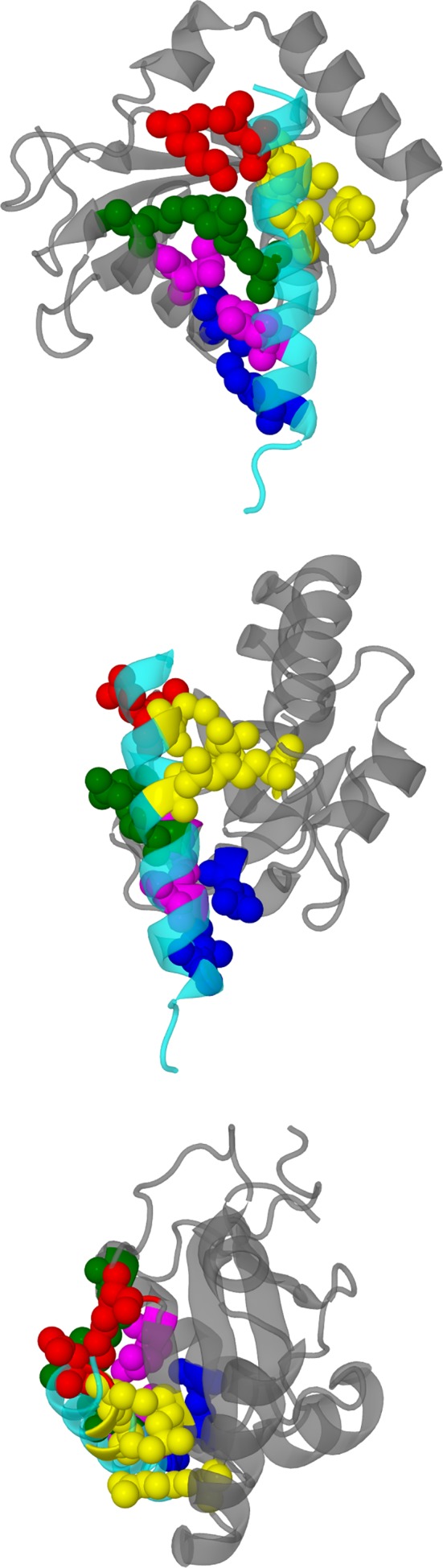
Diagrams comparing the key contact amino acid pairs between the intracellular helix of the *γ*2‐subunit of the GABA_A_ receptor and GABARAP. The intracellular helix is shown in cyan, while GABARAP is shown in gray. The contact amino acid pairs are divided into five groups, each group color coded in the following manner: (1) red ‐ *γ*2‐subunit Asp 423, GABARAP Lys 46 (2) yellow ‐ *γ*2‐subunit Cys 424 and Ala 428, and GABARAP Arg 28 and Leu 50 (3) green ‐ *γ*2‐subunit Cys 430 and GABARAP Arg 67 (4) magenta ‐ *γ*2‐subunit Ile 434 and GABARAP Leu 63 (5) blue ‐ *γ*2‐subunit Ile 438 and GABARAP Gln 59. The view of the top panel is from the ion channel towards the outside of the protein, that of the middle panel is from the side of the intracellular helix, and that of the lower panel is from the membrane towards the cytoplasm. These three views are roughly orthogonal to each other [Color figure can be viewed at wileyonlinelibrary.com]

**Figure 5:**
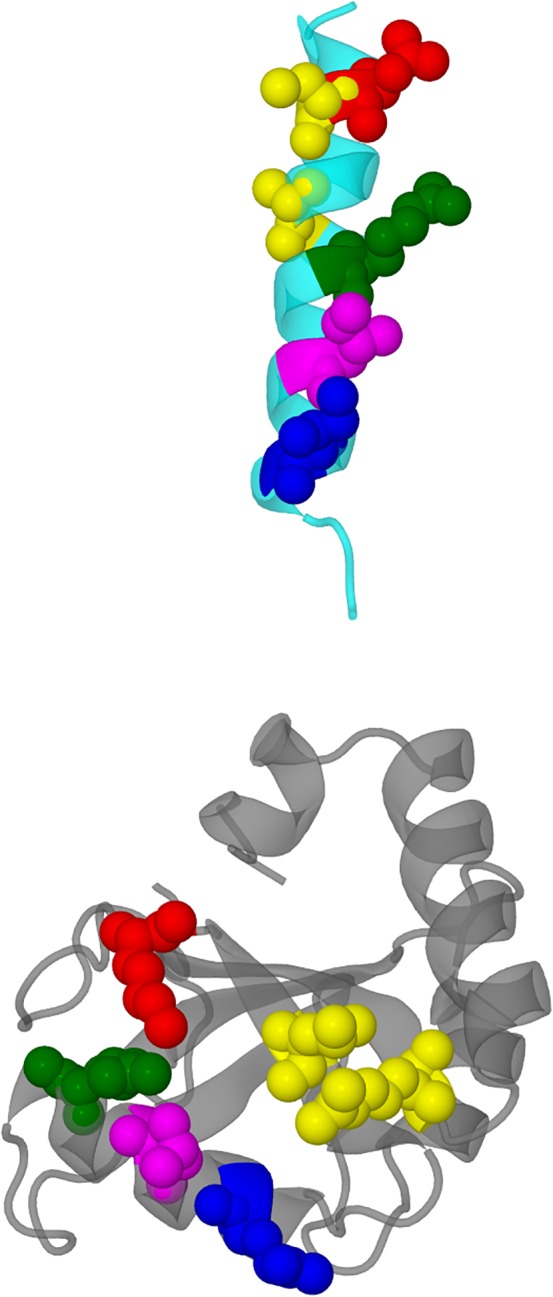
Diagrams comparing the key contact amino acid pairs between the intracellular helix of the *γ*2‐subunit of the GABA_A_ receptor and GABARAP. The intracellular helix is shown in cyan, whilst GABARAP is shown in gray. The contact amino acid pairs are divided into five groups, each group color coded in the following manner: (1) red ‐ *γ*2‐subunit Asp 423, GABARAP Lys 46 (2) yellow ‐ *γ*2‐subunit Cys 424 and Ala 428, and GABARAP Arg 28 and Leu 50 (3) green ‐ *γ*2‐subunit Cys 430 and GABARAP Arg 67 (4) magenta ‐ *γ*2‐subunit Ile 434 and GABARAP Leu 63 (5) blue ‐ *γ*2‐subunit Ile 438 and GABARAP Gln 59. The top panel shows the amino acids on the intracellular helix, and the bottom panel shows the amino acids on GABARAP [Color figure can be viewed at wileyonlinelibrary.com]

These contacts can be roughly grouped into five and their contact positions are shown in Figure 4. We also display the two contact faces individually in Figure 5. Experimental NMR research showed that GABARAP Lys 46, Val 51, Phe 60, and Ile 64 exhibited large shifts in their NMR spectrum on binding to the octadecapeptide R^425^TGAWRHGRIHIRIAKMD^442.^ 3 Yeast assays^1^ and fluorescence titration experiments^4^ showed that, in the tricosapeptide C^420^FEDCRTGAWRHGRIHIRIAKMD^442^, the amino acids RTGAW and GRIHIRIAKMD at both ends were of particular importance. Our docking results show that GABARAP Lys 46 is in contact with Asp 423 of the *γ*2‐subunit of the GABA_A_ receptor in all 11 docks, but we are unable to observe large contacts between GABARAP Val 51, Phe 60 and Ile 64. However, there are large contact areas in the neighboring amino acids: *γ*2‐subunit Cys 424 and Ala 428 both make contact with GABARAP Leu 50 in all 11 docks, *γ*2‐subunit Ile 438 makes contact with GABARAP Gln 59 in all 11 docks, and *γ*2‐subunit Ile 434 makes contact with GABARAP Leu 63 in all 11 docks. In addition, *γ*2‐subunit His 431 makes contact with GABARAP Leu 63 in 10 out of 11 docks, and *γ*2‐subunit His 435 makes contact with GABARAP Gln 59 in 10 out of 11 docks.

### 3.2 Simulation of GABARAP and intracellular helices

**Figure 6:**
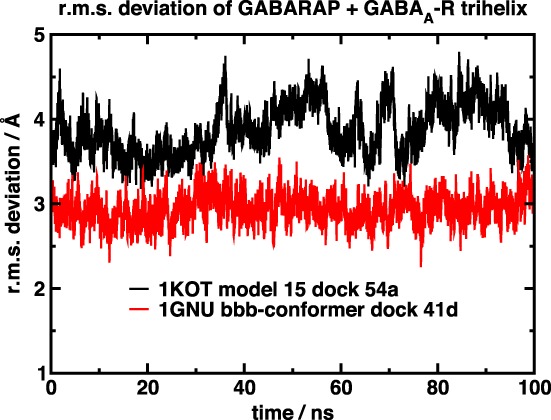
Diagram showing the r.M.S. deviation of the simulated structures from the starting structure during the 100‐ns data collection period [Color figure can be viewed at wileyonlinelibrary.com]

**Figure 7:**
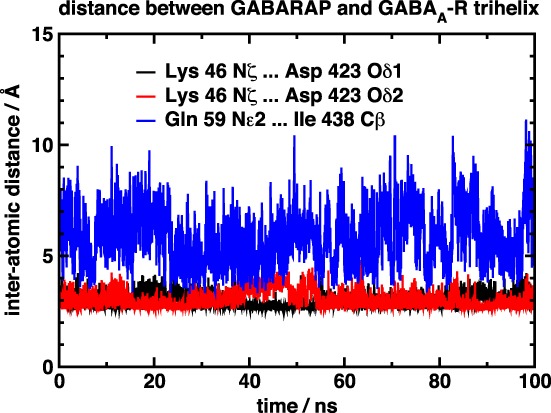
Diagram showing the distance between three atom pairs between GABARAP and the GABA_A_ receptor. The GABARAP configuration used was from 1KOT, model 15, dock 54a [Color figure can be viewed at wileyonlinelibrary.com]

**Figure 8:**
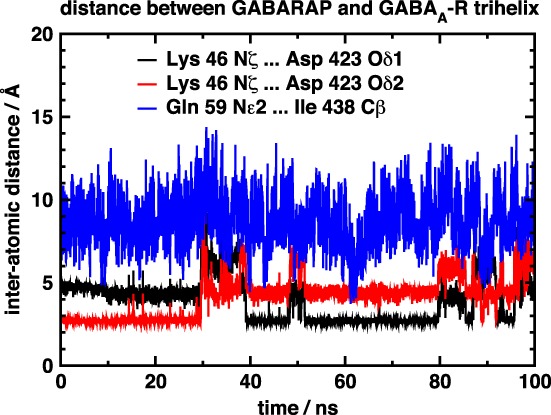
Diagram showing the distance between three atom pairs between GABARAP and the GABA_A_ receptor. The GABARAP configuration used was from 1GNU, bbb‐conformer, dock 41d [Color figure can be viewed at wileyonlinelibrary.com]

The r.m.s. deviation of the simulated structures is shown in Figure 6. The 1GNU structure shows a slightly higher r.m.s. deviation than the 1KOT structure, but the deviations remain stable throughout the simulation. The distances between the key amino acids are shown in, respectively, Figures 7 and 8. In both the 1KOT and 1GNU simulations, the distance between Lys 46 and Asp 423 is shorter than that between Gln 59 and Ile 438, and the latter also shows less variation than the former. We can rationalize this observation by noting that Lys 46 and Asp 423 are both charged, whereas Gln 59 is a polar amino acid and Ile 438 is a nonpolar one. In the 1GNU simulation, the distance between Lys 46 and Asp 423 atoms were generally below 5 Å, but sometimes it increased to above 10 Å. Visual inspection of the structures show that, in the case of the larger distances, the main chain of GABARAP has moved further away from the *γ*2‐subunit intracellular helix and there is a dihedral angle change in the side chain of Lys 46. All this can cause the N*ζ*‐atom of Lys 46 to move by as much as 5 Å.

It can be seen that GABARAP interacts in a stable manner with the GABA_A_ receptor intracellular helices.

### 3.3 Hydration of the GABA_**A**_ receptor intracellular domain

**Figure 9:**
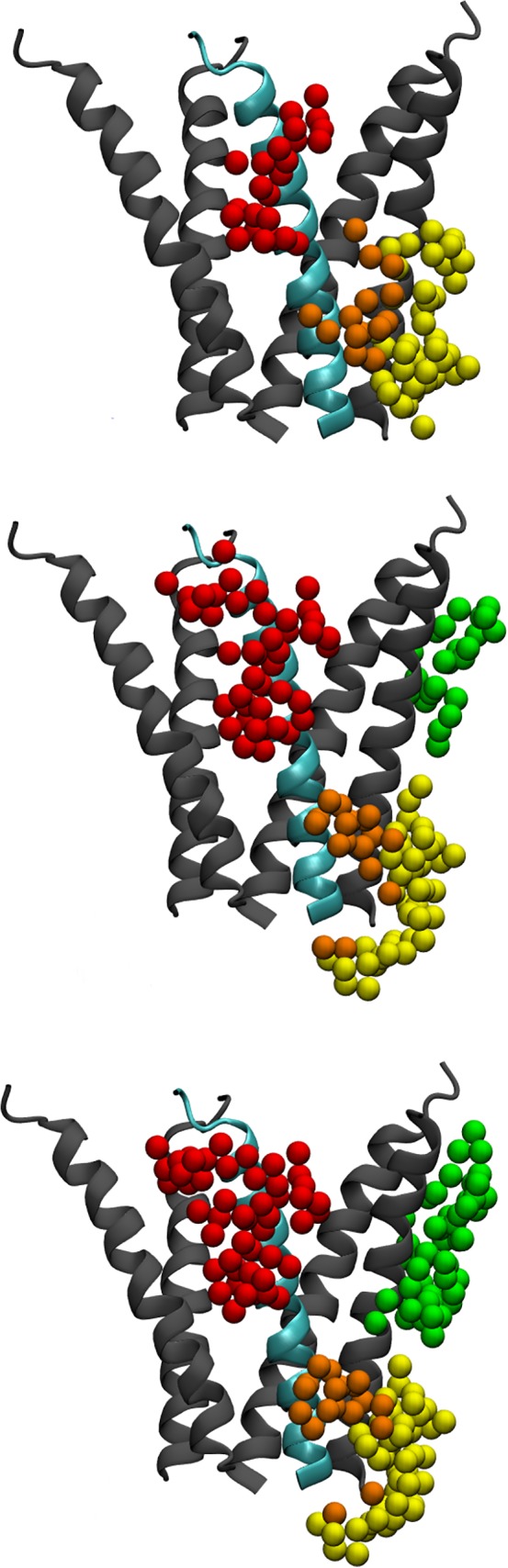
Diagram showing a model of the intracellular helices of the GABA_A_ receptor; the *γ*2‐subunit is shown in cyan. In the top panel, the hydration sites from the best “close” clusters of sizes 7‐18 as red, orange and yellow spheres. In the middle panel, the “regular” clusters are shown, while in the bottom panel, the “far” clusters are shown. The hydration sites are shown in color as described in Table 3 [Color figure can be viewed at wileyonlinelibrary.com]

The top panel of Figure 9 shows the most displaceable “close” hydration sites near the intracellular domain of the *γ*2‐subunit of the GABA_A_ receptor. It can be seen that there is a clustering of hydration sites on the *γ*2‐subunit as well as hydration sites on the adjacent *β*2‐subunit. The middle panel shows the most displaceable “regular” hydration sites near the intracellular domain of the *γ*2‐subunit of the GABA_A_ receptor. There is a similar clustering of hydration sites on the *γ*2‐subunit as well as hydration sites on the adjacent *β*2‐subunit including an additional higher patch. The bottom panel shows the most displaceable “far” hydration sites near the intracellular domain of the *γ*2‐subunit of the GABA_A_ receptor. The clustering of hydration sites on the subunits is similar to the “regular” case.

**Figure 10:**
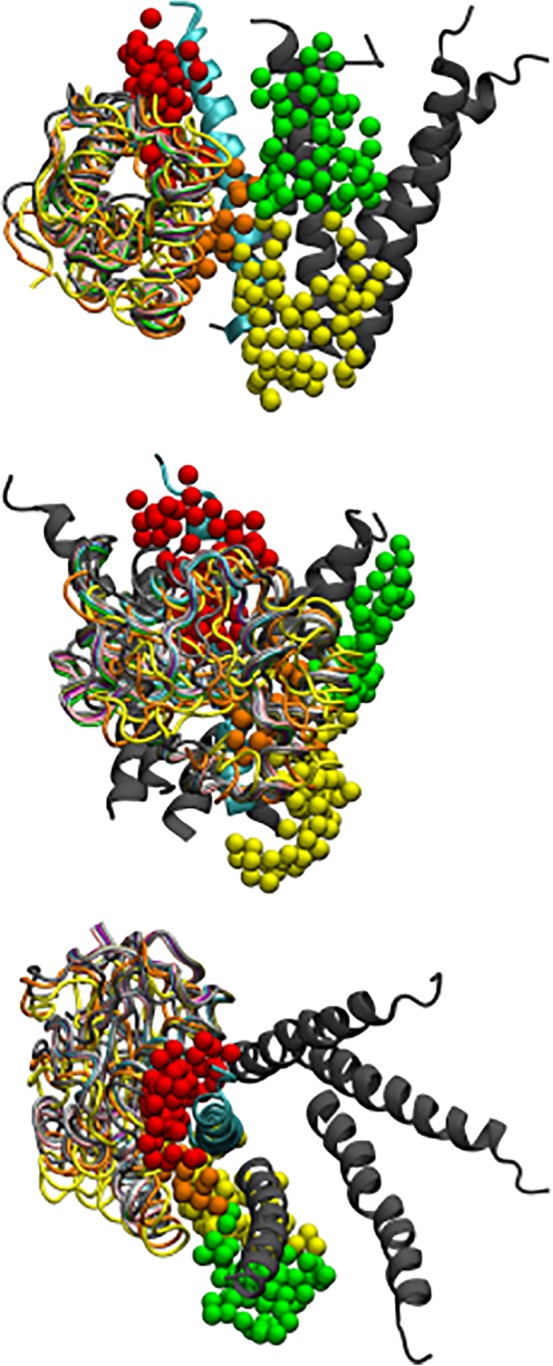
Diagrams comparing the overlaid main chains of predicted docking positions of GABARAP (multiple colors), and all the hydration sites (red, orange, yellow, green) identified in this work from “close,” “regular,” and “far” searches. The *γ*2‐subunit is shown in cyan. The hydration sites are shown in color as described in Table 3 [Color figure can be viewed at wileyonlinelibrary.com]

Figure 10 compares the hydration sites location with the location of the predicted SwarmDock poses. The GABARAP positions are very close to the red and orange hydration sites. It can be seen that there is considerable agreement between the predicted docked poses of GABARAP, and the identified hydration sites which could form the interface between the *γ*2‐subunit of the GABA_A_ receptor and GABARAP.

The three classes of hydration site clustering, “close,” “regular,” and “far” all show a set of most displaceble clusters: those primarily situated on the *γ*2‐subunit (red), those between the *γ*2 and *β*2‐subunits (orange) and those on the lower, cytoplasmic portion of the *β*2‐subunit (yellow). In addition to this a patch was found on the *β*2‐subunit (green) in the “regular” and “far” classes. As can be seen in Table 3, the red patch on the *γ*2‐subunit is the easiest to displace on average across all classes.

**Table 3:** Table of displacement statistics for clusters of GABA_A_ receptor hydration sites featuring in the top set. The units of all statistics are in kJ/mol except the number of patches combined to make the patch. The patches are those displayed in Figure 9

Patch	Mean	Median	Std dev	Number
Close (red)	−36.8	−39.0	5.0	244
Close (orange)	−40.5	−41.4	2.1	113
Close (yellow)	−40.8	−41.6	1.8	238
Regular (red)	−31.4	−38.8	13.6	246
Regular (orange)	−41.2	−41.5	0.8	76
Regular (yellow)	−40.1	−41.1	2.5	308
Regular (green)	−40.8	−41.2	1.0	119
Far (red)	−35.0	−40.6	11.1	252
Far (orange)	−40.5	−41.1	1.5	70
Far (yellow)	−38.0	−40.2	5.6	365
Far (green)	−39.4	−41.3	4.1	294

The amino acids within 5 Å of the red patch, in order of highest contact to lowest contact (name followed by frequency), are listed in Table 4. The tricosapeptide C^420^FEDCRTGAW**RH**GR**IH**IR**I**AK**M**D^442^ is required for full interaction, and all of these amino acids are found near the hydration sites (the bold amino acids are of greater importance in the interaction). For example, Met 441 is not found in the “close” binding but has increasing impact over distance from the protein. This amino acid may help influence GABARAP binding at far distances. Arg 430 is more contacted at close distances; this may help GABARAP bind once it is close.

**Table 4:** The frequency of amino acids within 5 Å of the most displaceable (red) patch. The amino acids in bold are of particular importance in this interaction

Name	Close	Regular	Far
Cys 420	8	0	0
Asp 423	4	0	0
Cys 424	8	1	1
Gly 427	5	5	6
Ala 428	9	8	8
**Arg 430**	19	13	13
**His 431**	17	17	17
Gly 432	1	1	1
**Ile 434**	17	16	16
**His 435**	16	17	17
Ile 436	9	9	9
Arg 437	1	1	13
**Ile 438**	17	17	18
Ala 439	0	5	5
Lys 440	0	0	2
**Met 441**	0	15	17
Ser 443	0	6	2

### 3.4 GABARAP hydration

**Figure 11:**
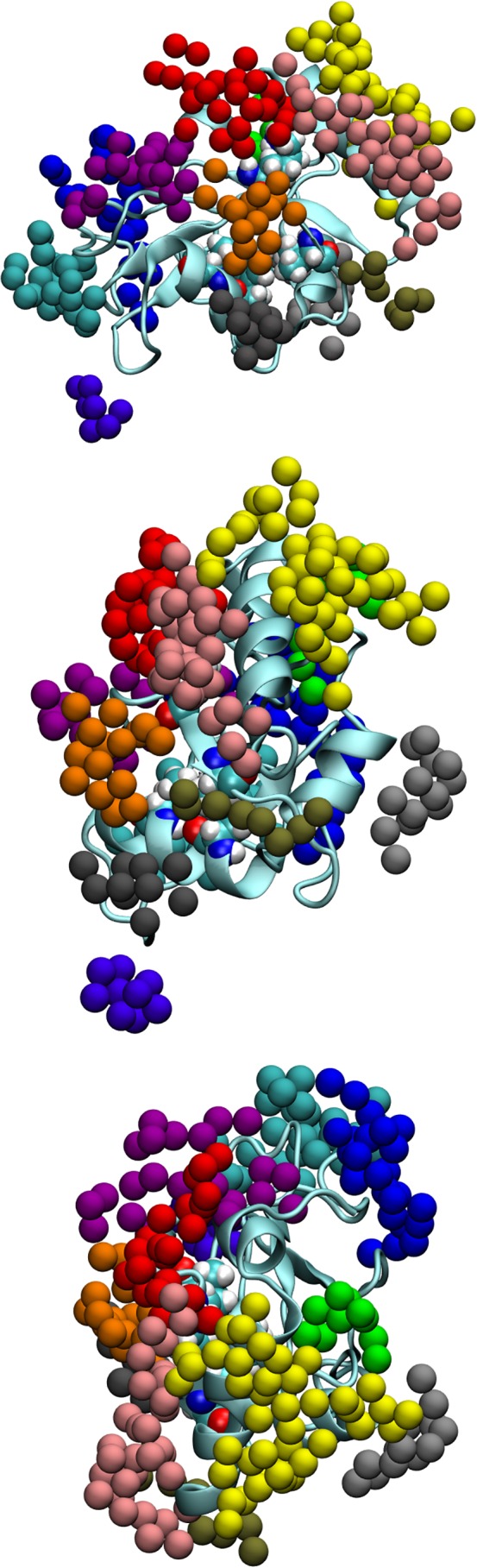
GABARAP with the hydration sites listed in Table 5. The CPK‐colored atoms are from residues Lys 48, Val 51, Phe 60, and Ile 64. The angles of view of these three panels are approximately the same as those for the three panels in Figure 3 [Color figure can be viewed at wileyonlinelibrary.com]

Table 5 and Figure 11 show the location of the main hydration patches on the surface of GABARAP. It is useful to divide these patches up into two: those with known binding proteins and those without. We define two kinds of hydration sites, “overlapping” sites where the hydration patch is directly over the binding face of the protein, and “surrounding” sites where the hydration patch is near the binding face of the protein. Note that these GABARAP hydration sites are different from the GABA_A_ receptor hydration sites but some of them share the same color codes.

**Table 5:** A guide to the locations of the hydration patches on GABARAP. Residues within 3 Å are listed. The mean displacement free energy (in kJ/mol) of hydration sites at that site and the number of hydration sites in the patch. The numbers under the sections K (1KOT) and G (1GNU) indicate which pass of the hydration site search these regions are highlighted. The sites are displayed in Figure 11

Name	Color	GABARAP nearby residues (≤3Å)	Mean	*N*_hs_	*K*	G
11	Red	D45 E8 E17 H9 K13 K47 K48 Y5	6.1	24	1	1
12	Purple	A36 A39 R67 D43 D45 E34 G42 I41	8.8	23	1	1(2)
		L44 K35 K2 K47 F3 Y115 Y5 V4				
13	Green	R14 D102 E100 H99 L105 K6 F104	11.3	10	1	(1)3
		F11 Y106				
21	Blue	N82 I84 L117 K2 K38 M1 P37 S113	6.8	25	2	1(4)
		Y115 V114				
22	Cyan	A75 R40 D111 D74 E112 G116 L117	6.7	25	2	1
		S110 V114				
31	Yellow	R15 R22 E101 E12 E19 K13 K23	4.5	42	3	(1)34
		F103 F11 P10 16				
32	Pink	D27 E17 I21 K13 K20 K24 K48	4.5	31	3	(1)234
		P26 Y25				
33	Orange	R67 D45 L50 L63 K46 K66 Y49	5.5	16	3	2(4)
41	Tan	R28 D27 P52	5.4	8	4	2(4)
42	d. Gray	L63 K66 F62	3.8	10	4	24
43	Silver	Q93 E97	3.8	13	4	23
51	Mauve	E73	3.1	9	5	‐

Table 6 shows the hydration patches involved in binding to known proteins, and the patches probably involved in GABARAP oligomerisation. The GABA_A_ receptor *γ*2‐subunit binds GABARAP with site 33 (orange) as the overlapping site, and sites 11 (red) and 12 (purple) as the surrounding sites.^2^, ^3^, ^4^ Calreticulin probably binds to two hydrophobic pockets^26^; for hydrophobic pocket 1, the overlapping site is site 32, and the surrounding site is site 33. For hydrophobic pocket 2, the overlapping site is site 33, and the surrounding site is site 42. The key GABARAP amino acids involved are Ile 21, Tyr 25, Ile 32, Lys 46, Lys 48, Tyr 49, Leu 50, Phe 60, and Leu 63 (PDB dataset 3DOW). The ALFY dodecapeptide^27^ binds to GABARAP overlapping sites 32 and 33, and surrounding site 11 (PDB dataset 3WIM). The KBTBD6 undecapeptide^28^ binds to GABARAP overlapping sites 11, 32, and 33, and surrounding sites 12 and 41 (PDB dataset 4XC2). The K1 dodecapeptide^5^ binds to GABARAP overlapping sites 32, 33, 41, and 42 and surrounding site 11 (PDB dataset 3D32). From the data from Coyle et al*.,* 4 we also suggest that site 43 is involved in GABARAP dimerisation. Lastly, the key tubulin‐binding amino acids in GABARAP are residues 10‐22. Tubulin binds GABARAP with sites 13, 31, and 32 as the overlapping sites, and site 11 as the surrounding site.

**Table 6:** Dictionary of hydration patches used for protein–protein interactions from PDB files related to GABARAP. The first half of the table lists the interaction between GABARAP and another protein, with the relevant PDB dataset or relevant publication shown in parenthesis. The second half of the table lists the interaction between GABARAP molecules (“self‐interaction”) in any oligomer; the relevant PDB dataset or relevant publication is listed with the chains involved. Parentheses () around a site number means it is partial. [other] indicates that lots of the amino acids are not near a hydration patch

GABARAP binding another protein		
Protein	Overlapping sites	Surrounding sites
GABA_A_ ‐R *γ*2‐subunit	11 32 33 41 42	
Calreticulin hp‐1 (3DOW)	32 (11)	33
Calreticulin hp‐2 (3DOW)	33	42 (12)
ALFY peptide (3WIM)	32 33 (41) (42)	11 (12)
KBTBD6 (4XC2)	11 32 33	12 41 (13) (31) (42)
K1 (3D32)	32 33 41 42 (12)	11 (13) (31)
Tubulin (26)	13 31 32	11
GABARAP “self‐interaction”		
PDB chain(s)	Overlapping sites	Surrounding sites
Dimerisation (4)	43	
4XC2 AC	42	(33)
4XC2 AD‐BC	21 22	(12)
4XC2 CA	[other, weak]	
4XC2 CB	32 (33)	(31)
4XC2 DA	32 33	11 12 41
4XC2 AH‐BG	21 22	12
4XC2 CE	[other]	(42)
3D32 AB	[other] 22 51	21
3D32 AD	[other] 21 22 51	
3D32 BA	32 41	31 (11) (33)
3D32 BD	[other] 32 33 42	11 12 41
3D32 BC	21 22	

There are a large number of hydration sites not involved in the binding of these three proteins. However, when we examine the crystallographic datasets, we find that these sites are involved in dimerisation or trimerisation. It is still unknown how GABARAP dimerises in the cell, so it is uncertain if these crystallographic oligomers represent the natural state of oligomerisation. Table 6 also shows the sites involved in GABARAP‐GABARAP interfaces (“self‐interaction”). Note that Coyle et al^4^ suggested a dimerisation face for GABARAP, but since no related PDB dataset has been reported, we have deduced the overlapping site from Figure 1 of the paper by Coyle et al.^4^ Moreover, the dimerisation suggested involves the N‐terminal amino acids “swinging out” to produce the “open” form of GABARAP; this “open” form of GABARAP is in a dimer form, and also simultaneously binds tubulin and the GABA_A_ receptor. We have access only to structural data of the “closed” form of GABARAP so the overlapping site identity is less certain than other sites.

### 3.5 Summary

**Figure 12:**
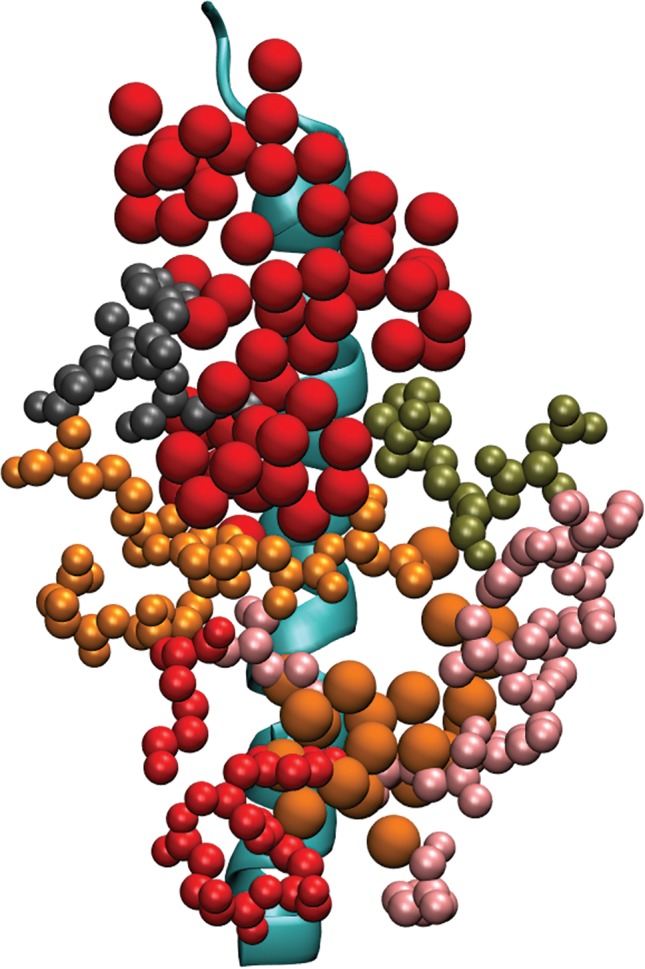
Diagram showing the GABA_A_ receptor red and orange hydration sites on the surface of its *γ*2‐subunit. In this diagram, the GABA_A_ receptor “close,” “regular” and “far” red hydration sites, as described in Table 3 are combined to give the red sites, and the “close,” “regular,” and “far” orange sites are combined to give the orange sites. The GABARAP residues are colored to correspond to their nearest sites according to the convention in Table 5: GABARAP sites 11, 32, 33, 41, and 42 are involved in this interaction [Color figure can be viewed at wileyonlinelibrary.com]

**Figure 13:**
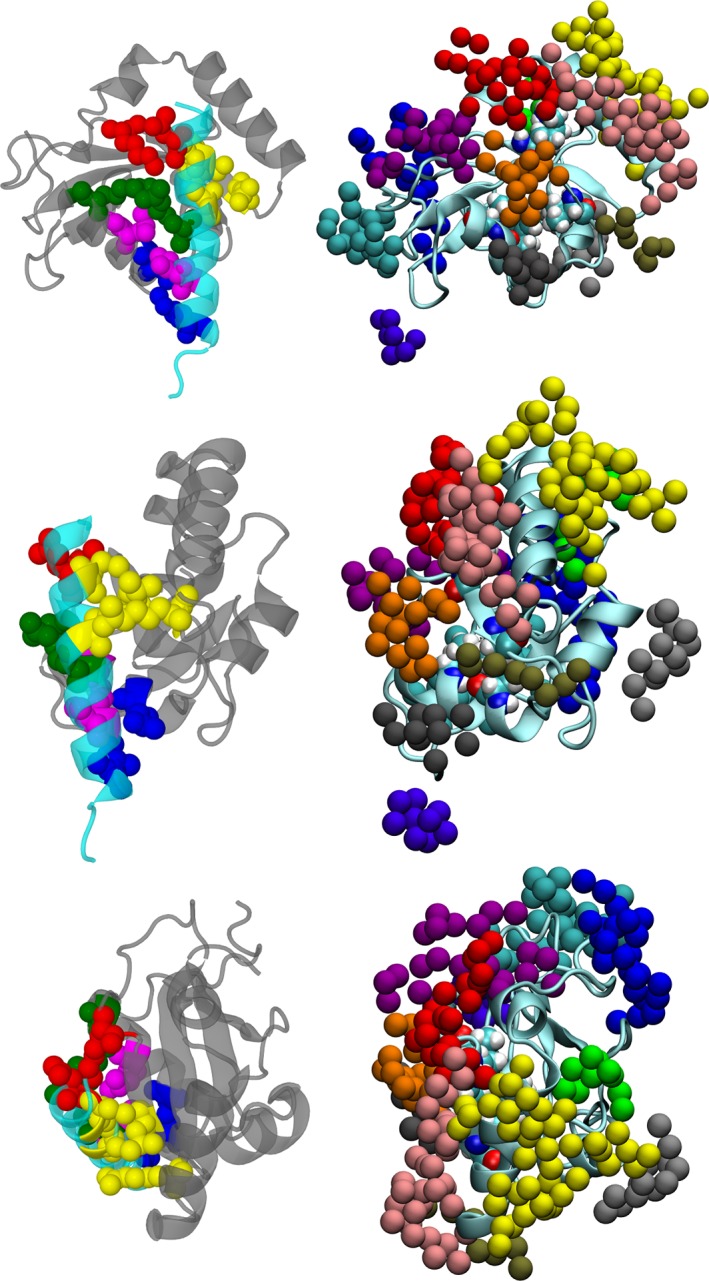
GABARAP with the hydration sites listed in Table 5. The CPK‐colored atoms are from residues Lys 48, Val 51, Phe 60, and Ile 64. The three panels on the left show the docking results with the intracellular MA helix of the *γ*2‐subunit of the GABA_A_ receptor present (transparent blue), and the three panels on the right show the results from hydration patch analysis. The angles of view on each row for the two structures are identical

Using SwarmDock and subsequent filtering based on available experimental evidence, we have identified 11 docked poses of GABARAP. These docked positions are all very similar, and they are all in contact with highly displaceable GABA_A_ receptor hydration sites. We note that the GABA_A_ receptor amino acids in Table 4 match those in Table 2 very well. Hydration analysis of water molecules around GABARAP has identified a large number of possible binding sites, and some of them are found to match the binding face for the GABA_A_ receptor *γ*2‐unit intracellular domain (see Figure 12). Figure 13 shows a global comparison of the results from docking and from hydration patch analysis.

However, in both cases, we have discovered hydration patches that might suggest a binding site, but we could not find any known binding molecule. In the case of the GABA_A_ receptor intracellular domain, there are hydration patches next to the *β*2‐subunit (green and yellow patches in Figure 9) which are distant from the GABARAP‐binding site, and do not seem to bind any known protein. In the case of the GABARAP, we have discovered hydration patches which suggest binding sites, but we could not find any protein that binds. Some of the GABARAP hydration patches are involved with binding tubulin, calreticulin, and various other peptides, though there is some degree of overlap between the GABA_A_ receptor binding site and the site for other proteins. It is interesting to note that the first‐pass and third‐pass sites are often involved in binding autophagy‐related proteins, but the second‐pass sites are used for dimerisation and trimerisation under crystallography conditions. Figure 11 also shows the hydration patches classified around GABARAP. The hydration patches from the 1GNU structure do not exactly match those from the 1KOT structure; the patches are defined by the 1KOT structure. Nevertheless, Table 5 shows that the first‐pass and second‐pass sites around 1KOT and 1GNU are very similar. Moreover, all the possible locations for hydration are identified in both cases, though they appear at different passes.

## 4 DISCUSSION

Cys‐loop ligand‐gated ion channels often interact with cytoplasmic proteins, and this interaction serves many purposes, amongst them the clustering of ion channels and the modulation of channel function.

One of the best studied examples is the interaction between the nAChR and the cytoplasmic protein rapsyn. Rapsyn has a molecular weight of about 43 000,^29^ and it interacts with the intracellular domain of the nAChR.^30^ Electron microscopy showed that the nAChR are interconnected by rapsyn dimers. Up to three rapsyn dimers can contact each nAChR in specific regions in the nAChR intracellular domain. This tight network probably underlies the low mobility of nAChR in the plane of the cell membrane, and also allows nAChR to be concentrated at the neuromuscular junction motor end‐plate.^30^

The interaction between the glycine receptor and gephyrin has been studied experimentally. Gephyrin was first identified as a protein which bridged the glycine receptor and tubulin.^31^ Sola et al.^32^ cocrystallized a segment of the glycine receptor *β*‐subunit and a partial dimer of the cytoplasmic protein gephyrin (Protein Data Bank code: 1T3E). They were able to resolve the structure of a pentapeptide portion of the glycine receptor *β*‐subunit and the gephyrin domain E dimer. They proposed a network of gephyrin molecules linking the glycine receptors. Unfortunately, only the structure of five amino acids of the receptor was resolved, so it is difficult to draw any conclusion from this dataset.

Gephyrin also interacts with the GABA_A_ receptor through its *α*2‐subunit^33^ and *α*3‐subunit.^34^ It is unclear if gephyrin binds the *α*1‐subunit of the GABA_A_ receptor; some experiments failed to show any interaction,^35^ but others showed a weak interaction.^36^ Maric et al.^37^ co‐crystallized segments of the *α*3‐subunit of the GABA_A_ receptor with segments of gephyrin, and identified the undecapeptide T^367^FNIVGTTYPIN^381^ from the GABA_A_ receptor as important for interaction with gephyrin. They showed that there were similarities between the binding of the GABA_A_ receptor and of the glycine receptor to gephyrin: T^367^FNIVGTT^374^ from the GABA_A_ receptor, and F^398^SIVGSL^404^ the glycine receptor *β*‐subunit adopted similar conformations.

Two other cytoplasmic proteins are known to interact with the GABA_A_ receptor: collybistin and GABARAP. Collybistin consists of two types, which consist of 413 and 493 amino acids, respectively.^38^ Saiepour et al^35^ showed that collybistin interacted with the intracellular domain of the *α*2‐subunit of the GABA_A_ receptor, and its binding site for the *α*2‐subunit overlapped that for gephyrin. Collybistin was later shown to be important for clustering gephyrin and the GABA_A_ receptor.^39^

GABARAP is a protein of 117 amino acids,^1^ and it binds specifically to the *γ*2‐subunit of the GABA_A_ receptor. Coyle et al.^4^ showed that GABARAP also binds tubulin, and this is believed to position the synaptic GABA_A_ receptors correctly in the membrane. Binding of GABARAP to the GABA_A_ receptor caused receptor clustering,^6^, ^7^ so some of its functions are similar to gephyrin and collybistin. However, GABARAP is unique in that its binding also caused the conductance of the GABA_A_ receptor to increase from about 30 pS to 40 pS‐60 pS, and the mean opening times from about 2 ms to about 6 ms.^40^ It thus appears that gephyrin has more general actions on both the GABA_A_ receptor and the glycine receptor, and that the action of gephyrin and collybistin appear to be confined to receptor clustering. The action of GABARAP is more specific to the GABA_A_ receptor, and, in addition to receptor positioning, it also modulates the electrophysiology of this ion channel.

In this work, we have used a flexible protein‐protein docking programme to identify the interaction between the GABA_A_ receptor and GABARAP. We have also used a novel method to predict hydration sites on the two proteins, and suggest docking poses. We have identified possible binding faces on the GABA_A_ receptor and on GABARAP. To confirm our theoretical predictions would require a high‐resolution structure of the GABA_A_ receptor with an intact intracellular domain.

Some of the GABARAP binding faces we have identified are at the GABARAP/GABA_A_ receptor interface, but others are involved in binding other proteins. In addition, we have also identified possible faces not known to bind any protein. It is interesting to note that, in the case of GABARAP, hydration patches appear on five out of six faces of this protein. As so many interfaces are involved in different types of interaction, it is possible that the last face is not active to remove the burden of constraints on protein architecture.

Currently, this method only examines the hydration details around proteins. We could envisage including details such as shape and electrostatic properties, and develop a molecular docking method based on this hydration site survey.

The GABA_A_ receptor in neurons have different ion channel properties from recombinant receptors.^41^ Luu et al.^40^ and Everitt et al.^7^ show that GABA_A_ receptor conductances in neurons is similar to that obtained from recombinant receptors associated with GABARAP. GABARAP is thus of importance in physiological functioning of the GABA_A_ receptor in the central nervous system, and this underlies the importance of understanding the physiological role of the intracellular domain of this receptor. It would be interesting to investigate the interaction between GABARAP and the GABA_A_ receptor further, to understand how GABARAP changes the ion channel functioning of the receptor. This would require a high‐resolution structure of the GABA_A_ receptor with an intact intracellular domain.

## CONFLICT OF INTERESTS

The authors declare that they have no conflicts of interest.

## Supporting information


**Appendix S1**: Supporting Information.
